# Comparative Evaluation of the Mechanical and Physical Properties of Mineral Trioxide Aggregate vs. Bacterial Cellulose Nanocrystal-Reinforced Mineral Trioxide Aggregate: An In Vitro Study

**DOI:** 10.7759/cureus.63632

**Published:** 2024-07-01

**Authors:** Lalitha Priya V, Kavitha Ramar

**Affiliations:** 1 Department of Pediatric and Preventive Dentistry, SRM Kattankulathur Dental College and Hospital, SRM Institute of Science and Technology, Chennai, IND

**Keywords:** scanning electron microscope, microhardness, compression strength, mineral trioxide aggregate, bacterial cellulose nanocrystals

## Abstract

Aim: This study aims to compare and assess the compression strength, microhardness, and surface texture of two sets of materials: mineral trioxide aggregate (MTA) Plus^TM^ and bacterial cellulose nanocrystal (BCNC)-reinforced MTA Plus^TM^.

Materials and methods: According to the ASTM E384 standard, the cylindrical molds made of plexiglass with an internal diameter of 6 mm and a height of 4 mm were fabricated using computer numerical control laser cutting. A total of 20 samples (n=10) in each group were considered in this experimental study: Group I (control group) MTA Plus^TM ^(Prevest DenPro Limited, India) and Group II (experimental group) BCNC (Vedayukt India Private Limited, India)-reinforced MTA Plus^TM^. After preparation, the molds were incubated at 37°C in a fully saturated condition for about 24 hours, and then the compression strength, microhardness, and scanning electron microscopy analyses were performed at different magnifications. The obtained data were then statistically analyzed.

Results: Quantitative analysis revealed that there is a statistically significant difference between MTA Plus^TM ^and BCNC-reinforced MTA Plus^TM^^ ^(p<0.002). The Wilcoxon signed-rank test and Mann-Whitney U-test revealed that BCNC-reinforced MTA Plus^TM^^ ^showed significantly higher compression strength (33.80±3.83 MPa, p=0.00) and surface microhardness (642.85±24.00 μm, p=0.00) than the control group.

Conclusion: Based on our findings, it was concluded that there is a statistically significant difference between both study groups. Thus, incorporating BCNC into the MTA Plus^TM^^ ^significantly increased the compression strength and surface microhardness of the MTA Plus^TM ^cement.

Clinical significance: Numerous dental applications have been investigated for bacterial cellulose. Many benefits of bacterial cellulose are available, which include its effects on moldability, low cost, high water retention capacity, biocompatibility, and biodegradability. Furthermore, the addition of BCNC to MTA Plus^TM^^ ^accelerates the material's hardening process and decreases its setting time, which in turn shortens clinical chairside procedural timing and thereby improves patient satisfaction.

## Introduction

The primary challenge in contemporary restorative dentistry is to encourage the remineralization of less mineralized carious dentine, thereby protecting and preserving the tooth pulp. Dentinal remineralization involves restoring minerals by producing inorganic mineral-like materials. Partial caries removal techniques with a biological basis have been advocated to prevent carious pulp exposure. Recent consensus findings suggest that the complete or selective eradication of all caries is now considered overtreatment. In restorative dentistry, bioactive materials are defined as those that form a surface layer of an apatite-like material in the presence of an inorganic phosphate solution. These materials have significantly improved restorative dentistry by enhancing the longevity of restorations and reducing the incidence of recurrent caries. Additionally, by exchanging fluorides, these chemical compounds can now remineralize the tooth structure [1-2]. Hence, bioactive materials such as mineral trioxide aggregate (MTA) and bacterial cellulose were included in the study.

Lee et al. were the first to introduce MTA in dental literature, stating that it is a mixture of bismuth oxide and Portland cement [3]. These hydraulic cement are formed through the hydration reaction between tricalcium silicate and dicalcium silicate. MTA was initially developed for clinical situations where maintaining a dry oral environment was challenging [4]. However, due to its beneficial properties such as biocompatibility, bioactivity, hydrophilicity, sealing capabilities, and minimal solubility, it has been widely adopted for various applications [4-5]. These include apexification, apexogenesis, pulpotomies, and as a material for pulp capping [5-7].

Despite its advantages, MTA faces clinical performance issues due to difficulties in manipulation, low compression strength, long setting time, high cost, and tooth discoloration [8]. To address these limitations, researchers have worked on modifying hydraulic cement using substances such as methylcellulose, calcium chloride, zinc oxide, eugenol, and chitosan [9]. Notably, the addition of bacterial cellulose nanocrystals (BCNCs) has enhanced the physiochemical properties of MTA.

MTA and biodentine are both commonly used in endodontics and restorative dentistry, but MTA is often considered superior. MTA has a longer history and more extensive clinical evidence supporting its effectiveness and long-term success. It is highly biocompatible and bioactive, promoting the formation of hard tissue barriers and the healing of pulp and surrounding tissues. Additionally, MTA's superior radiopacity makes it easier for clinicians to identify and assess radiographs, which is crucial for monitoring treatment success. It also has inherent antimicrobial properties, reducing the bacterial load and enhancing the healing process.

Cellulose is derived from readily available sources, such as plant-based materials and certain types of bacteria [9]. Bacterial cellulose is an eco-friendly polymeric material and naturally occurring hydrogel that is gaining attention. This unbranched polymer, composed of (1→4) β-glycosidic linked glucose units, features nanofibrils produced by specific aerobic bacteria, primarily from the *Komagataeibacter* genus. Within this genus, *Komagataeibacter xylinus* is the most notable species responsible for this synthesis by utilizing glucose as a substrate [10-12]. Bacterial cellulose offers certain advantages, such as interconnected porosity, excellent water-holding capacity, superior biocompatibility, and ease of shaping, with mechanical properties similar to those of some hard and soft tissues [11]. It is non-toxic, hydrophilic, non-allergenic, and biodegradable when modified, making it valuable in both the medical and dental fields.

Additionally, bacterial cellulose has received "generally recognized as safe" (GRAS) status for food use, approved by the US Food and Drug Administration [13-14]. This material was developed by the Council of Scientific and Industrial Research, Indian Institute of Integrative Medicine, and was classified under code 42 in the DCGI for dental materials [14]. Thus, it has excellent potential as a biomaterial for dental and oral applications and can be combined with various dental materials.

The microhardness and compressive strength of MTA are important attributes for evaluating the quality and progression of the hydration process. Therefore, the current research aimed to compare and assess the compression strength, microhardness, and surface texture of two sets of materials: MTA Plus^TM^ (Prevest DenPro Limited, India) and BCNC (Vedayukt India Private Limited, India)-reinforced MTA Plus^TM^. The objective was to investigate whether the natural BCNCs could serve as cost-effective, accessible alternatives to MTA Plus^TM^, potentially enhancing its quality.

## Materials and methods

This laboratory study was conducted in March 2023 at the Department of Pediatric and Preventive Dentistry at SRM Kattankulathur Dental College and Hospital, Chennai, India. It received approval from the SRM Scientific and Institutional Review Board (approval number: SRMIEC-ST0323-1301).

Type of study

This was a controlled laboratory study designed to compare and assess the physical properties (compression strength, microhardness, and surface texture) of two materials: MTA Plus^TM^ and BCNC-reinforced MTA Plus^TM^. The experimental setup allowed for precise quantification and comparison of these effects.

Sample size determination

The sample size was determined as 20 samples based on calculations using G*Power software (Heinrich-Heine-Universität Düsseldorf, Düsseldorf, Germany) targeting a 95% power and a 5% margin of error, leading to the inclusion of 20 samples.

Sample preparation

A cylindrical mold made of plexiglass with an internal diameter of 6 mm and a height of 4 mm, fabricated according to the ASTM E384 standard, was created using a computer numerical control (CNC) laser cutter. This process was carried out under optimal conditions (power: 4200 watts, cutting speed: 4400 mm/min, and 0.8 Mpa) at Saveetha Institute of Dental Science and Research, Chennai, India. The mold was used for compression strength and microhardness testing.

For this study, bacterial cellulose was utilized under 21 CFR 182.1, proving it to be GRAS as a food ingredient. This designation complies with the Federal Food, Drug, and Cosmetic Act 201(s) (21 USC Section 321(s)), achieved through scientific methods and the independent conclusion process. This status ensures the safety and non-toxicity of bacterial cellulose, making it a suitable component for dental applications. Cement manipulation of BCNC-reinforced MTA Plus^TM^ was a 3:1 water-to-powder ratio, while MTA Plus^TM^ was carried out according to the manufacturer’s instructions, thereby using a standard 1:1 powder-to-water ratio. The slurry was prepared by vigorously mixing the two components for about 30 seconds at a temperature of 23±2°C [15].

To ensure precise and accurate results, the samples were prepared under controlled laboratory conditions. The BCNCs were carefully integrated into MTA Plus^TM^ to form a homogeneous mixture. To preserve its properties, it is advisable to store the prepared mixture in a brown bottle in a cool, dark place, protected from direct sunlight. Each sample was then placed into the cylindrical molds and allowed to be set under standardized conditions. Following the setting period, the samples were subjected to compression strength testing using a universal testing machine. The microhardness of the samples was measured using an atomic force microscopy (AFM) tester.

The combination of CNC laser-fabricated molds and the rigorous testing process aimed to provide reliable data on the enhanced properties of MTA Plus^TM^ when reinforced with BCNCs. This meticulous approach ensured that the findings were robust and could be confidently compared to the traditional MTA Plus^TM^ material. Hence, a total of 20 samples (n=10 per group) were considered in this experimental study. The samples were prepared and tested as per standardized procedures to ensure the validity and reliability of the results. The groups analyzed were as follows: Group I (control group) MTA Plus^TM^ and Group II (experimental group) BCNC-reinforced MTA Plus^TM^.

Scanning electron microscope analysis

After cement placement into the mold, a total of 20 samples (n=10 per group) were incubated at 37°C and 97% relative humidity in a fully saturated condition for about 24 hours. Then the samples were removed from the molds. After solidification, the specimens were affixed to a sample holder coated with a carbon film. Observations were conducted using a scanning electron microscope (SEM) at specific magnifications (500X, 1000X, and 2000X).

Microhardness test

**Figure 1:**
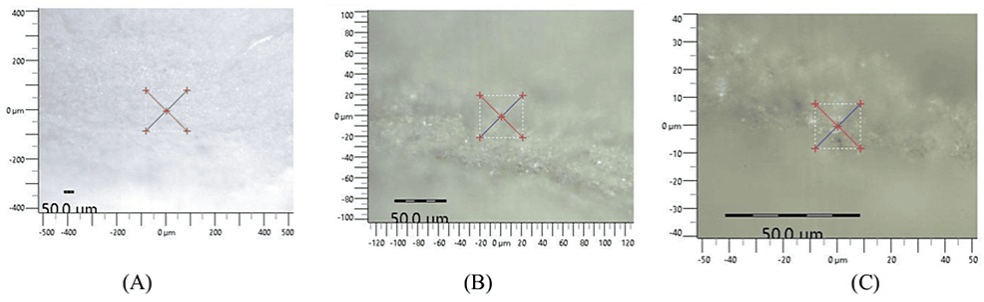
Images of AFM tester creating indentations for evaluating microhardness at (A) 5x magnification, (B) 20x magnification, and (C) 50x magnification AFM: atomic force microscopy

To measure hardness, the sample surfaces were polished using 400-2000 grit silicone carbide sandpaper. The samples were subsequently tested using an AFM tester equipped with a pyramid-shaped diamond indenter, applying a force of 300 grams for approximately 10 seconds. Three distinct indents were created on the polished surface of each specimen. These indentations were positioned at a distance from each other and from the sample's edge equal to 2.5 times the diameter of the indents, and the microhardness values were calculated, as seen in Figure 1.

Assessment of compression strength

The samples were further placed lengthwise between the plates of the universal testing machine for compression at a rate of 1 mm per minute, with the fracture load being measured in megapascals (MPa). The compressive strength values were calculated by following the equation: RC = F x 9.807/A, where RC is the compressive strength (MPa), F is the force/unit area (kg), and A is the base area.

Statistical analysis

SPSS Statistics version 16.0 (SPSS Inc., Released 2007; SPSS for Windows, Version 16.0; Chicago, SPSS Inc.) was used for statistical analysis. The mean values and standard deviations for compressive strength and microhardness were calculated. The Wilcoxon signed-rank test and Mann-Whitney U test were employed for evaluating compressive strength and microhardness, with a significance level set at p<0.002. Additionally, Z-tests were conducted to determine the significance of the observed differences between mean values and standard deviations. To ensure a conservative estimation of the p-value, tests such as asymptotic significance (2-tailed) and exact significance (2*(1-tailed Sig.)) were also performed.

## Results

Comparative data on three key mechanical properties (maximum force, compression strength, and microhardness) of the two dental materials is provided in Table 1. The results indicate the following: in terms of maximum force, MTA Plus^TM^ exhibited a higher maximum force (mean=282.40 N, p=0.00) compared to BCNC-reinforced MTA Plus^TM^ (mean=190.87 N, p=0.00). With regards to the compression strength, BCNC-reinforced MTA Plus^TM^ showed significantly higher compression strength (mean=33.80 MPa, p=0.00) compared to MTA Plus^TM^ (mean=22.31 MPa, p=0.00). Correspondingly, BCNC-reinforced MTA Plus^TM^ had a significantly higher microhardness (mean=642.85 μm, p=0.00) compared to MTA Plus^TM^ (mean=434.50 μm, p=0.00).

**Table 1:** Mean and standard deviation of MTA PlusTM and BCNC-reinforced MTA PlusTM MTA Plus^TM^: mineral trioxide aggregate Plus^TM^, BCNC-reinforced MTA Plus^TM^: bacterial cellulose nanocrystal-reinforced mineral trioxide aggregate Plus^TM^, maximum force (N): maximum force represented as newtons, compression strength (MPa): compression strength represented as megapascals, microhardness test (μm): microhardness test represented as micrometers, statistical significance is represented as p-value (p≤0.00)

Material	Maximum force (N)		Compression strength (MPa)		Microhardness test (μm)	
	Mean	Standard deviation	Mean	Standard deviation	Mean	Standard deviation
BCNC-reinforced MTA Plus^TM^	190.87	27.56	33.80	3.83	642.85	24.00
MTA Plus^TM^	282.40	56.51	22.31	6.83	434.50	10.71
p-value	p≤0.00		p≤0.00		p≤0.00	

This visual representation supports the numerical data previously provided, reinforcing the potential benefits of using BCNC-reinforced MTA Plus^TM^ in dental applications where compressive strength and microhardness are crucial.

**Figure 2:**
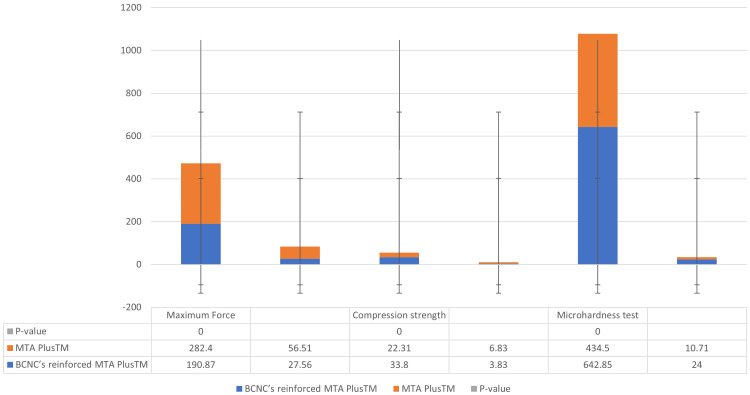
Bar graph representing compressive strength, microhardness, and maximum force of MTA PlusTM and BCNC-reinforced MTA plusTM materials Statistical significance is represented as (*p<0.02) vs. MTA; (#p<0.02) vs. BCNCs modified. MTA Plus^TM^: mineral trioxide aggregate Plus^TM^, BCNC’-reinforced MTA Plus^TM^: bacterial cellulose nanocrystal-reinforced mineral trioxide aggregate Plus^TM^, maximum force (N): maximum force represented as newtons, compression strength (MPa): compression strength represented as megapascals, microhardness test (μm): microhardness test represented as micrometers, error bars indicate the standard deviation for each measurement

In Figure 2, the comparison of various mechanical properties between MTA Plus^TM^ and BCNC-reinforced MTA Plus^TM^ is expressed. For maximum force, MTA Plus^TM^ (represented by the blue bar) has a mean value of 282.4 N, while BCNC-reinforced MTA Plus^TM^ (represented by the orange bar) shows a mean value of 190.87 N. In terms of compressive strength at maximum force, MTA Plus^TM^ has a mean of 33.8 MPa, whereas BCNC-reinforced MTA Plus^TM^ has a mean of 22.31 MPa. Finally, the microhardness test reveals that MTA Plus^TM^ has a mean of 642.85 μm, compared to BCNC-reinforced MTA Plus^TM^, which has a mean value of 434.5 μm.

In addition to the Wilcoxon signed-rank test and Mann-Whitney U test, Z-scores are also negative for compression strength and surface microhardness (-3.480 and -3.024, respectively), which strongly indicates that there are statistically significant differences between both study groups.

**Figure 3:**
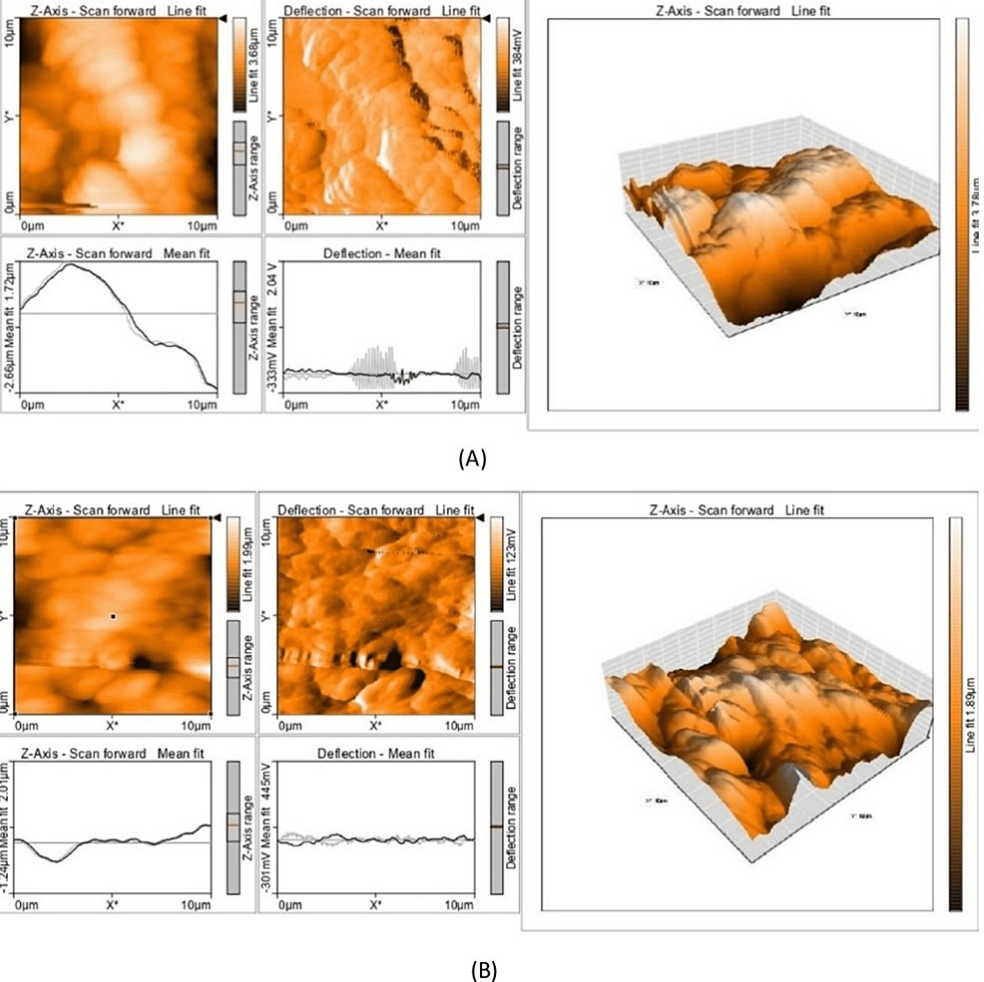
3D image of microhardness evaluated using an AFM tester: (A) MTA PlusTM and (B) BCNC-reinforced MTA PlusTM μm: micrometer unit, mV: millivolt unit, V: volt unit, 3D image: three-dimensional image, Z-axis: average height, AFM: atomic force microscopy, MTA Plus^TM^: mineral trioxide aggregate Plus^TM^, BCNC-reinforced MTA Plus^TM^: bacterial cellulose nanocrystal-reinforced mineral trioxide aggregate Plus^TM^

The two sets of graphs in Figures 3A-3B present share several common elements but also exhibit distinct differences. Both sets include a top-left image titled "Z-axis - Scan Forward Line Fit," which shows a surface topographical map in the Z-axis direction. These images use color gradients to represent height variations, with a similar spatial resolution of 0 µm to 10 µm. Additionally, both sets include a feature at the top middle image titled "Deflection - Scan Forward Line Fit," displaying deflection maps during the forward scan. The color gradients in these images indicate different levels of deflection, with scales showing the deflection range.

Both sets also include graphs in the bottom left and bottom middle positions. The bottom left graph, titled "Z-axis - Scan Forward Mean Fit," plots the average height (Z-axis) across a line scan over a distance of 10 µm. The lines in these graphs represent height profiles, showing variations in surface height along the scanned line. The bottom middle graph, titled "Deflection - Mean Fit," shows average deflection values across the same line scan distance. These lines represent mean deflection profiles, indicating changes in deflection along the scanned line. Lastly, both sets of graphs feature a right-side 3D representation of the surface topography.

Despite these commonalities, there are notable differences between the two sets of graphs. In Figure 3A, the Z-axis range appears less pronounced with smoother variations in height. In contrast, Figure 3B shows more prominent peaks and valleys, indicating a rougher surface. Henceforth, BCNC-reinforced MTA Plus^TM^ displays more distinct and irregular variations.

The AFM of MTA Plus^TM^ shows a peak reaching around 1.72 µm, whereas the AFM of BCNC-reinforced MTA Plus^TM^ displays a peak reaching approximately 2.01 µm and a lower trough at around -1.42 µm, indicating greater variability. The bottom middle deflection profile in Figure 3A shows a range of approximately -2.34 V to 2.04 V, indicating a smoother transition with fewer pronounced features, while Figure 3B indicates a range from around -301 mV to 45 mV, revealing a more rugged surface with distinct peaks and valleys.

**Figure 4:**
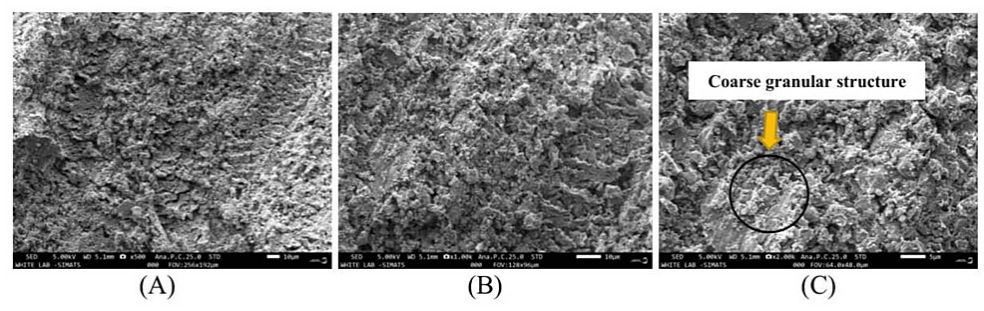
MTA PlusTM scanning electron micrographs at magnifications of 500X (A), 1000X (B), and 2000X (C) where irregular and porous structures can be observed MTA Plus^TM^: mineral trioxide aggregate Plus^TM^

To understand the presumptive reasons and surface textures of both groups, we analyzed the samples under SEM. SEM images of MTA Plus^TM^ samples in Figure 4 revealed irregular structures with pores of more or less different shapes and sizes, and loose granules were observed.

**Figure 5:**
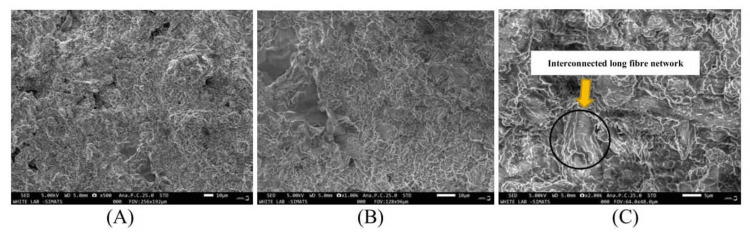
BCNC-reinforced MTA PlusTM SEM at magnifications of 500X (A), 1000X (B), and 2000X (C) where minimal porous structure with increased crystallinity can be observed BCNC-reinforced MTA Plus^TM^: bacterial cellulose nanocrystal-reinforced mineral trioxide aggregate Plus^TM^, SEM: scanning electron microscope

On the other hand, SEM analysis for BCNC-reinforced MTA Plus^TM^ in Figure 5, evaluated at 500x, 1000x, and 2000x magnifications, revealed that BCNC-reinforced MTA Plus^TM^ exhibits a less porous structure with increased crystallinity and that the chemical processing did not affect the original structure of bacterial cellulose.

## Discussion

MTA Plus^TM^ is extensively utilized in endodontics and restorative dentistry due to its excellent properties. However, it presents certain drawbacks such as high cost, extended setting time, difficulty in manipulation, and precise placement due to its granular consistency, potential for discoloration, sensitivity to moisture, and difficulty in removal once set if retreatment or adjustments are needed. Despite these disadvantages, MTA Plus^TM^ remains favored for its biocompatibility, sealing ability, and bioactive properties.

To address these drawbacks, incorporating bacterial cellulose into MTA Plus^TM^ may offer a superior alternative. Bacterial cellulose holds significant promise as a biomaterial for dental and oral applications, being mainly used in dental pulp tissue regeneration, periodontal regeneration, and surgical wound dressings for the oral mucosa. Bacterial cellulose boosts properties such as porosity control, high water retention capacity (up to 98 wt%), low production cost, excellent mechanical strength due to hydrogen bonds between fibrillar units, non-immunogenicity, and hemocompatibility. In a dynamic culture, it can produce hydrogels in forms such as cocoons, pellets, and irregular shapes. Incorporating bacterial cellulose with MTA Plus^TM^ will expedite its hardening process, thus reducing its setting time and, consequently, the clinical chairside procedure duration.

The current research is the first of its kind to evaluate the physiochemical properties of MTA Plus^TM^ modified with a novel hydrophilic nanopolymer material. Strength is generally defined as the stress required to fracture a material. Compressive strength is assessed by applying a compressive load to a cylindrical specimen, with measurements varying based on the type of occlusal load applied. Physiochemical properties, such as compression strength, are essential for hydraulic cement when subjected to occlusal forces [16]. Surface microhardness plays a vital role in achieving an ideal seal. On the other hand, masticatory forces produce heavy occlusal loads, which may lead to displacement of the restorative material and disruption of the physical seal. Due to the strong correlation between a material's microhardness and its setting response, any delay in the setting of MTA Plus^TM ^could potentially lead to a reduction in its microhardness [17-18].

In this comparative study, MTA Plus^TM^ demonstrated significantly higher maximal force resistance than BCNC-reinforced MTA Plus^TM^, indicating superior mechanical resilience, potentially favorable for applications demanding robustness. The statistical analysis with a p-value of 0.000220 confirms the presence of a significant difference, effectively rejecting the null hypothesis. Nonetheless, the variability observed in MTA Plus^TM^, as evidenced by its higher standard deviation and mean, may compromise its clinical predictability.

Similarly, in 2022, Pushpalatha et al. highlighted that MTA Plus^TM ^has faced long-standing technical challenges related to setting time, mechanical properties, color stability, handling characteristics, and its paste-like consistency [4]. Effective hydration reactions between the water and the powder components in hydraulic cement are closely associated with the physical properties, notably the compression strength [19-20]. Thus, alterations in the powder-to-water ratio between these hydraulic cements lead to a reduction in the compression strength. Also, Basturk et al. demonstrated that elevating the water-to-powder ratio leads to a reduction in the compressive strength of MTA Plus^TM^ [19]. Therefore, in this current study, compression strength was measured over a 24-hour period, and hence the obtained results revealed a statistically significant difference between both groups. As a result, the incorporation of BCNCs into MTA Plus^TM^ (mean±SD is 33.80±3.83 MPa, p<0.002) significantly increased the compression strength values of the cement in comparison to MTA Plus^TM^ (mean±SD, 22.31±6.83 MPa, p<0.002).

Conversely, BCNC-reinforced MTA Plus^TM^ showed superior average compressive strength relative to MTA Plus^TM^, an attribute critical in restorative dentistry for materials exposed to masticatory forces. The lower standard deviation of BCNC-reinforced MTA Plus^TM^ suggests a more consistent performance, enhancing its predictability in clinical applications. The statistical significance of this finding, revealed by a p-value of 0.000204, supports the enhanced reliability of BCNC-reinforced MTA Plus^TM^, where high compressive strength is paramount. Also, the BCNC-reinforced MTA Plus^TM^ samples reported significantly higher microhardness values in contrast to the control group (mean±SD is 642.85±24.00 μm vs. 434.50±10.71 μm, respectively).

In this experimental study, to analyze the surface texture of MTA Plus^TM^ and BCNC-reinforced MTA Plus^TM^, SEM was performed. MTA Plus^TM ^typically exhibits a porous, granular structure in SEM analysis, which can complicate its manipulation and affect its setting time. On the other hand, the SEM results of BCNC-reinforced MTA Plus^TM^ exhibit a less porous structure with a high amount of acicular hydrosilicates [15]. Acicular hydrosilicates are minerals that form elongated, needle-like crystals and are characterized by their thin, elongated crystal habit resembling needles or fibers. These hydrosilicates present in bacterial cellulose can accelerate the setting reaction of MTA Plus^TM^ by serving as nucleation sites for hydration. Their needle-like structure increases the surface area available for the chemical reactions during MTA Plus^TM^'s setting process, thereby speeding up the overall reaction rate. These hydrosilicates also promote the formation of a more extensive and interconnected network of hydration products, leading to a quicker and more complete setting process. Also, the acicular shape enhances the mechanical interlocking of MTA Plus^TM^ particles, contributing to a more stable and faster-setting matrix. Consequently, incorporating bacterial cellulose containing acicular hydrosilicates reduces the overall setting time of MTA Plus^TM^, making it more efficient for clinical applications where time is of the essence.

Additionally, the BCNCs have the capacity to form hydrogen bonds with the hydroxyl groups present in the tricalcium silicate as well as the carboxylic groups of the water-soluble polymer. This interaction is a potential explanation for the observed enhancement in its mechanical properties. The incorporation of BCNCs into MTA Plus^TM^ may lead to a broader range of particle size distributions [9]. Consequently, these small nanocrystals and fiber networks could fill the gaps between the larger tricalcium silicate particles, creating more binding sites for the liquid polymer and, in turn, reinforcing the MTA Plus^TM^ [9].

Prior research carried out by Voicu et al. demonstrated that the inclusion of BCNCs in MTA did not adversely affect cell viability. They incorporated BCNCs into MTA at two different ratios: MTA at 10% biocell and MTA at 33% biocell. The study found that adding bacterial cellulose to MTA maintained good crystallinity, suggesting that the chemical processing preserved the original structure of the bacterial cellulose. Cell morphology observations using both contrast phase and fluorescence microscopy showed that MTA+33% biocells did not adversely affect cell viability. Voicu et al. evaluated the biocompatibility, morphology, and structure of MTA cement reinforced with bacterial cellulose [15]. They assessed the binding properties, including setting time at 37°C and compressive strength, with samples prepared in accordance with the ISO 6876:2012 standard. Pastes were made using a cement-to-water weight ratio of 3:1, and the quartering method was applied at 20±2°C for 90 seconds. The study found that bacterial cellulose particles demonstrated high compatibility with the hydrocompounds formed during the cement hardening process, leading to improved characteristics, particularly in terms of setting time and biological properties.

Our study further supports these findings, as evidenced by the comparative data on BCNC-reinforced MTA Plus^TM^ and MTA Plus^TM^. The results show that BCNC-reinforced MTA Plus^TM^ exhibits a mean compression strength (33.80 MPa), which is significantly higher than MTA Plus^TM^ (22.31 MPa). This aligns with Voicu et al.'s findings regarding the enhanced structural integrity provided by bacterial cellulose incorporation. Additionally, the microhardness test results from our study indicate that BCNC-reinforced MTA Plus^TM^ (642.85 μm) has a higher mean value compared to MTA Plus^TM^ (434.50 μm). This suggests improved hardness and wear resistance, which is consistent with the observed high compatibility and enhanced characteristics mentioned by Voicu et al. Similarly, Recouvreux et al. and Lin and Dufresne noted that microbial cellulose exhibits a substantial water retention capacity, reaching as high as 98% of its weight [21-22].

There are certain limitations to our study. Our study focused on a detailed analysis of specific parameters that required intensive testing, including detailed SEM analysis and microhardness testing. This necessitated a smaller but carefully selected sample size to ensure rigorous analysis and accurate results. Hence, adequate statistical power analysis was conducted to ensure that the sample size was sufficient to meet the study objectives. Furthermore, SEM analysis provides detailed images and surface texture for both groups. Its limited depth of field may prevent accurate capture of the true 3D topography of the MTA Plus^TM^ cement surface. Additionally, SEM can potentially damage the MTA Plus^TM^ surface, especially when high-energy electron beams are employed, which can alter surface characteristics and compromise the accuracy of the analysis. Despite attempts to simulate oral cavity conditions, certain inherent variables in in vitro studies, such as constant temperature, pH, and static conditions, do not accurately mirror the natural fluctuations found in human oral environments. These differences may affect the compression strength and microhardness of MTA Plus^TM^ and BCNC-reinforced MTA Plus^TM^ agents under real-world conditions. Further, in vitro studies lack biological complexity, are performed under static conditions, and do not consider the immune response. Moreover, the physical forces acting on materials in the human body, such as pressure and shear stress, are not replicated in in vitro studies, which can affect the performance of materials and devices.

Although in vitro studies provide valuable preliminary data and insights, as a future scope, their results need to be validated through in vivo studies and clinical trials to ensure their relevance and effectiveness in real-world medical and dental applications [23]. To validate the observed superior properties and characteristics of BCNC-reinforced MTA Plus^TM^, we further recommend conducting more studies with larger sample sizes, assessing additional factors like biocompatibility, solubility, and color stability.

## Conclusions

Based on the results acquired from the current study, it is evident that the inclusion of BCNCs has a notable impact, leading to a significant increase in both the compression strength and microhardness of MTA Plus^TM^. This novel cement, crafted from MTA Plus^TM^ and BCNCs, exhibits intriguing attributes when compared to commercial products. In the context of employing MTA Plus^TM^ for vital pulp therapy, this innovative material, BCNC-reinforced MTA Plus^TM^, presents itself as a compelling alternative.
